# MiR‐339 inhibits proliferation of pulmonary artery smooth muscle cell by targeting FGF signaling

**DOI:** 10.14814/phy2.13441

**Published:** 2017-09-26

**Authors:** Jidong Chen, Xiaolei Cui, Li Li, Junle Qu, J. Usha Raj, Deming Gou

**Affiliations:** ^1^ Shenzhen Key Laboratory of Marine Bioresource and Eco‐environmental Science Shenzhen Key Laboratory of Microbial Genetic Engineering College of Life Sciences Shenzhen University Shenzhen Guangdong China; ^2^ Key Laboratory of Optoelectronic Devices Systems of Ministry of Education and Guangdong Province College of Optoelectronic Engineering Shenzhen University Shenzhen Guangdong China; ^3^ Department of Pediatrics University of Illinois at Chicago Chicago Illinois

**Keywords:** FGF2, FRS2, miR‐339, PASMC, PDGF‐BB, proliferation

## Abstract

Pulmonary artery hypertension (PAH) is a fatal disorder. Recent studies suggest that microRNA (miRNA) plays an important role in regulating proliferation of pulmonary artery smooth muscle cells (PASMC), which underlies the pathology of PAH. However, the exact mechanism of action of miRNAs remains elusive. In this study, we found that miR‐339 was highly expressed in the cardiovascular system and was downregulated by a group of cytokines and growth factors, especially PDGF‐BB and FGF2. Functional analyses revealed that miR‐339 can inhibit proliferation of PASMC. Also, miR‐339 inhibited FGF2‐induced proliferation, but had no effect on proliferation induced by PDGF‐BB. The fibroblast growth factor receptor substrate 2 (FRS2) was identified as a potential direct target of miR‐339. Consistent with the actions of miR‐339, knockdown of FRS2 only inhibited FGF2‐ but not PDGF‐BB‐induced proliferation of PASMC. In addition, our results showed that inhibition of ERK and PI3K abrogated the downregulation of miR‐339 induced by PDGF‐BB. Finally, miR‐339 expression was found to be decreased in the pulmonary arteries of rats with MCT‐induced PAH. Our study is the first report on the biological role of miR‐339 in regulating proliferation of PASMC by targeting FGF signaling, providing new mechanistic insights into PASMC proliferation and pathogenesis of PAH.

## Introduction

Pulmonary arterial hypertension (PAH) is a progressive disease of the lung vascular system, primarily affecting small pulmonary arterioles (Lai et al. 2014). Pathologically, PAH is a fast progressing vascular disease characterized by uncontrolled cell proliferation, migration, and reduced apoptosis of pulmonary artery smooth muscle cell (PASMC), leading to a progressive increase in pulmonary vascular resistance, right ventricular failure, and ultimately death (Stenmark et al. 2006; Lai et al. 2014). Although there has been some progress in the treatment of PAH patients, survival rates still remain exceedingly low (Lai et al. 2014). PAH has a multifactorial etiology and its incidence involves with various reactive gas molecules, growth factors, and cytokines such as platelet‐derived growth factor BB (PDGF‐BB), fibroblast growth factor 2 (FGF2), transforming growth factor beta (TGF‐*β*), angiotensin II (Ang II), and endothelin‐1 (ET‐1), and so forth (Saltis et al. 1992; Bayes‐Genis et al. 2000; Millette et al. 2006; Berghausen et al. 2013; Leung et al. 2013).

PDGFs are regarded as the most potent mitogen for PASMC, which bind and activate their receptors (PDGFR*α* and *β*). Activated PDGFR recruit and activate downstream effectors, regulating expression of target genes and biological processes such as proliferation and migration (Kazlauskas 1994; Heldin and Westermark 1999; Rosenkranz and Kazlauskas 1999). Increased PDGF signaling may contribute to the pathobiology of PAH (Raines 2004; Grimminger and Schermuly 2010). Elevated PDGF or PDGFR expression has been detected in lung tissue and small pulmonary arteries in experimental PAH animals (Arcot et al. 1993; Cai et al. 1996; Kwapiszewska et al. 2005; Jones et al. 2006) and patients with PAH (Humbert et al. 1998; Lanner et al. 2005). On the other hand, genetic ablation of PDGF‐dependent signaling pathways abolishes vascular remodeling and experimental pulmonary hypertension (Ten Freyhaus et al. 2015). Imatinib, a PDGF receptor antagonist, has been reported to improve PAH in animal models as well as in some patients (Berghausen et al. 2013; Gomberg‐Maitland et al. 2013). However, this was accompanied by severe adverse events and drug discontinuation was common (14, 40). FGF2 is another mitogenic signal factors implicated in PAH development by stimulating cell proliferation and survival in PASMC (Izikki et al. 2009). In a report, Benisty et al. (2004) studied 117 patients with PAH and 60 control subjects, and discovered that median levels of urinary and plasma bFGF were significantly higher in patients with PAH compared to normal control subjects; in another report, Mohamed and colleagues found endothelial FGF2 is overproduced in patients with idiopathic pulmonary hypertension (IPH) and MCT‐induced PAH, which contributed to SMC hyperplasia in PAH (Izikki et al. 2009). Moreover, inhibition of FGF signaling ameliorated the PAH condition (Zheng et al. 2015).

MicroRNAs (miRNAs) are a group of single‐stranded RNA molecules about 19–23 nucleotides in length (Lewis et al. 2005). Normally, miRNAs function by interacting with the 3ʹ untranslated region (UTR) of specific mRNA targets to repress translation or induce degradation post‐transcriptionally (Lewis et al. 2005). MiRNAs regulate gene expression and play significant roles in diverse cellular processes, including proliferation, migration, differentiation, and apoptosis (Cheng et al. 2015). In the past decade, miRNAs have been implicated in the biological function of PASMC and the development and progression of PAH (Barwari et al. 2016). For instance, miR‐210 has an antiapoptotic effect in PASMC during hypoxia (Gou et al. 2012), whereas miR‐328 promotes apoptosis (Guo et al. 2012). Additionally, a series of miRNAs, such as miR‐451, miR‐22, miR‐145, and miR‐322 have been reported to be expressed differentially in rat models of PAH induced by monocrotaline or chronic hypoxia (Caruso et al. 2010, 2012). In our previous report, miRNAs expressed in PASMC exposed to PDGF‐BB were investigated globally for the first time and a group of miRNAs were found to be expressed differentially in response to PDGF‐BB stimulation (Chen et al. 2016; Qian et al. 2016). Among these miRNAs, miR‐328 was downregulated by PDGF‐BB, a miRNA that inhibited proliferation and migration of PASMC by targeting PIM‐1 (Qian et al. 2016), whereas miR‐376b expression was increased by PDGF‐BB, a miRNA that increased proliferation of PASMC by targeting BMPR2 (Chen et al. 2016).

In this study, we found that miR‐339, one of the miRNAs regulated by PDGF‐BB identified in our earlier study, was highly expressed in cardiovascular system, which implicated a potential role for miR‐339 in cardiovascular diseases. We found that miR‐339 could be downregulated by many cytokines and growth factors, and that it inhibited the proliferation of PASMC by targeting FRS2 (fibroblast growth factor receptor substrate 2), a critical adaptor protein in the FGF signaling pathway (Chen et al. 2009).

## Methods

### Ethics statement

All experiments were carried out according to China Council on Animal Care and the protocols used were approved by the Animal Care and Use Committee of Shenzhen University, China.

### Cell culture and treatment

Human embryonic kidney (HEK) 293 cells were obtained from American Type Culture Collection (ATCC, Manassas) and maintained in Dulbecco's modified Eagle's medium (DMEM, Invitrogen) supplemented with 10% fetal bovine serum (FBS, Invitrogen). Rat PASMC (RPASMC) was isolated and cultured in DMEM supplemented with 10% FBS as reported previously (Zeng et al. 2015).

RPASMCs were starved for 12 h with DMEM supplemented with 0.2% FBS for 12 h and treated with cytokines and growth factors (ANGII [200 ng/mL], ET‐1 [25 ng/mL], FGF2 [30 ng/mL], IGF1 [30 ng/mL], PDGF‐AA [30 ng/mL], PDGF‐BB [30 ng/mL], TGF‐*β* [20 ng/mL], VEGF [30 ng/mL], and FBS [10%], R&D) for specific time periods (0 h, 2 h, 4 h, 6 h, 12 h, 24 h). For kinase inhibition studies, before being stimulated with growth factors, starved cells were pretreated with corresponding inhibitors (imatinib [5 *μ*mol/L], U0126 [10 *μ*mol/L], SH‐4‐54 [5 *μ*mol/L], enzastaurin [5 *μ*mol/L], pictilisib [1 *μ*mol/L], Selleck) for 0.5 h.

### MiRNA and siRNA transfection

Chemically synthesized mimic and inhibitor of miR‐339 for overexpression and suppression of endogenous mature miR‐339 expression, and the corresponding miRNA‐negative control (mimic and inhibitor NC) were provided by Ruibobio (Guangzhou, China). The miRNA mimic and inhibitor were transfected in a final concentration of 20 nmol/L and 50 nmol/L, respectively, using K2 transfection reagent (Biontex, Germany) as recommended by the manufacturer. SiRNA targeting FRS2 and si‐NC were transfected as miRNA mimic with a final concentration of 50 nmol/L.

### Cell proliferation assay

Cell proliferation was measured by EdU incorporation (EdU [5‐ethynyl‐2′‐deoxyuridine] is a thymidine analog and incorporated into DNA during active DNA synthesis), being carried out using the EdU Assay Kit (Ribobio) according to the manufacturer's instruction. The stained cells were examined using a fluorescent inverted microscope. Cell proliferation rate was calculated as the number of cells with EdU staining divided by the number of cells stained with DAPI. The MTS assay, as described previously (Wang et al. 2010), was used to assess cell viability with MTS Assay Kit (Promega) according to the manufacturer's instruction.

### Quantification of mRNA and miRNA expression

Total RNA was extracted with RNAiso Plus (Takara, Dalian, China) and quantified using the NanoDrop 2000c Spectrophotometer (Thermo Fisher Scientific). For mRNA expression detection, first strand cDNA was synthesized from 1 *μ*g of total RNA treated by DNase using oligo(dT) plus random hexamer primers with M‐MLV Reverse Transcriptase (Invitrogen). The quantitative PCR experiments were performed on Step‐One plus real‐time PCR System (Applied Biosystems) using SYBR green‐I Master PCR Mix with gene‐specific primers. The *β*‐actin was used as reference gene for normalization (Usui et al. 2014). Primers used for mRNA determination were listed in Table 1.

**Table 1:** Primers list

FRS2 forward primer	5ʹ‐AGCTGTCCAGATAAAGACACTGT‐3ʹ
FRS2 reverse primer	5ʹ‐ATTTTACCGAGTCCCGTTTCC‐3ʹ
*β*‐actin forward primer	5′‐AAAGACCTGTACGCCAACAC‐3′
*β*‐actin reverse primer	5′‐GTCATACTCCTGCTTGCTGAT‐3′
MiR‐339 RT primer	5ʹ‐GTGCAGGGTCCGAGGTCAGAGCCACCTGGGCAATTT TTT TTTTTCGTGAG‐3ʹ
MiR‐339 forward primer	5ʹ‐CCGGGTCCCTGTCCTCCAGGAG‐3ʹ
Sno202 RT primer	5ʹ‐GTGCAGGGTCCGAGGTCAGAGCCACCTGGGCAATTTTTT TTT TTCATCAG‐3ʹ
Sno202 forward primer	5ʹ‐GTA CTT TTG AAC CCT TTT CCAT‐3ʹ
FRS2‐UTR forward primer	5′‐CGGAATTCTATATGGCCTAAGAGTGCAA‐3′
FRS2‐UTR reverse primer	5′‐CCCTCGAGTGCATAGTACAGTATACGTC‐3′
FRS2‐UTR mut forward primer	5′‐AAAATGGCTGTCCCGCCTTACGTTCTCATAACTCC‐3′
FRS2‐UTR mut reverse primer	5′‐GTAAGGCGGGACAGCCATTTTTAACAGAACTTTAGT‐3′

The miRNA expression was determined using S‐Poly(T) Plus real‐time PCR method as detailed in our previous work (Niu et al. 2015), and snoRNA‐202 was used as the reference gene (Brattelid et al. 2011). The primer sequences used were listed in Table 1. The relative expression of mRNA and miRNA was calculated using the 2^−∆∆CT^ method.

### Western blotting

Cells were lysed with ice‐cold RIPA (50 mmol/L Tris‐HCl, pH 7.5, 150 mmol/L NaCl, 1% NP‐40, 0.25% sodium deoxycholate, 1 mmol/L EDTA) buffer supplemented with protease inhibitor cocktail (Sigma‐Aldrich). Each sample with 30 *μ*g protein was then electrophoresed on the sodium dodecyl sulfate (SDS) polyacrylamide gel and then electroblotted to nitrocellulose filter membranes (Millipore). Membranes were immersed in 5% degreased milk powder diluted in TBST (20 mmol/L Tris‐HCl, pH7.5, 100 mmol/L NaCl, 0.1% Tween‐20) for 1 h and incubated with antibodies against FRS2 (1:1000, Sanying, Wuhan, China), *β*‐actin (1:5000, Santa Cruz Biotechnology) overnight at 4°C. Next, the membranes were washed and incubated with horseradish peroxidase‐conjugated secondary antibodies (1:5000, Jackson Immuno‐Research) for 1 h at room temperature. The protein bands were visualized with the SuperSignal chemiluminescent detection module (Thermo Fisher Scientific).

### 3′ UTR luciferase reporter assay

TargetScan algorithm (http://www.targetscan.org) was applied to predict targets and the binding sites of miR‐339. The 3′ UTR of FRS2 was PCR amplified and inserted into pGL4‐plasmid (Promega). The corresponding mutant constructs with six mutated residues in the region of seeding sequence were generated by site‐directed mutagenesis. The primers used were listed in Table 1. Luciferase activity was measured in cell extracts with a Lumat LB9508 luminometer (Berthold, Germany). Cells were cotransfected with experimental firefly and control renilla luciferase constructs. Firefly luciferase activity was normalized to renilla luciferase activity to account for potential differences in transfection and/or cell lysis efficiency.

### Rat PAH model and measurement of hemodynamics

PAH was induced in adult male Sprague Dawley rats (four per experimental group) by a single intraperitoneal injection of monocrotaline (MCT, 60 mg/kg, Sigma) and then studied 4 weeks later. To measure the right ventricular pressure, rats were initially anesthetized with an intraperitoneal injection of pentobarbital sodium (30 mg/kg) and placed on a heated table to maintain their temperature during the procedure. The right jugular vein was surgically exposed, and a 1.2‐Fr pressure catheter connected to AP‐621G (Nihon Kohden) was inserted in the right ventricle (RV) through the incision in the right jugular vein. The right ventricular systolic pressure (RVSP) was recorded using MP150 system and AcqKnowledge software package (BIOPAC). To assess right ventricular hypertrophy, saline containing 5 U/ml heparin was flushed into the RV after death and the heart removed. The RV wall was separated from the left ventricular (LV) wall and the ventricular septum (S). The ratio of the weight of the RV wall and that of the LV wall plus ventricular septum (LV+S) was used to assess right ventricular hypertrophy.

### Statistical analysis

All data shown are mean values with standard deviation (SD) of at least three experiments. When only two groups compared, different significance was assessed with the double‐sided Student's *t* test. Different significance between groups were analyzed using one‐way ANOVA. A *P* < 0.05 was considered significant.

## Results

### MiR‐339 expression is decreased in MCT‐induced PAH rats

**Figure 1:**
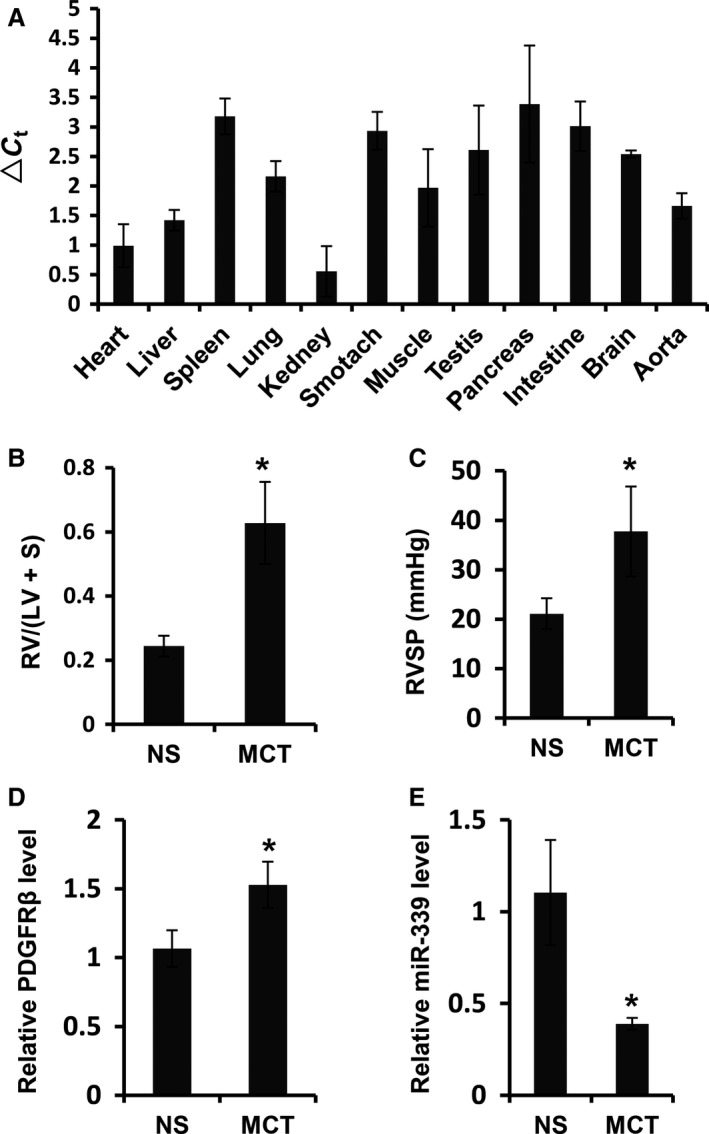
MiR‐339 expression was decreased in pulmonary arteries of MCT‐induced PAH rats. (A) Histogram showed the miR‐339 expression in various organs in rats, ▵Ct = Ct(miR‐339)‐Ct(sno202), *n* = 5. (B) The ratio of right ventricle (RV) and left ventricle (LV) plus ventricular septum (S) RV/(LV+S) in MCT‐induced rats was elevated significantly (*n* = 4) compared with that in the controls (*n* = 4). (C) Right ventricular systolic pressure (RVSP) in MCT‐induced rats was elevated significantly (*n* = 4) compared with that in the controls (*n* = 4). (D) Expression of PDGFR *β* mRNA in pulmonary arteries of MCT‐induced rats was increased significantly (*n* = 4) compared with that in the controls (*n* = 4). (E) Expression of miR‐339 in pulmonary arteries of MCT‐induced rats was decreased significantly (*n* = 4) compared with that in the controls (*n* = 4). MCT represented MCT‐treated rats, NS represented normal saline‐treated rats; different significance was assessed with Student's *t* test, *P < 0.01.

Recently, we have identified 13 miRNAs that were differentially expressed in rat PASMC (RPASMC) in response to PDGF‐BB stimulation (Chen et al. 2016). The expression level of these miRNAs were measured in different organs of the rat and one miRNA, miR‐339, was found to be relatively highly expressed in the arteries, compared to most other organs (Fig. 1A), implying the importance of miR‐339 in cardiovascular diseases. A recent report showed that miR‐339 in exosomes can affect function of VSMC (Tan et al. 2016), however, little is known about its expression and function in PASMC.

To investigate whether miR‐339 may play a role in PAH development, we further detected the expression of miR‐339 in the pulmonary arteries of MCT‐induced PAH rat. Development of PAH was evident in rats 3 weeks after MCT administration as a significant increase in the right ventricle to left ventricle + septum ratio compared to normal saline‐treated rats (Fig. 1B), together with an increase in right ventricular systolic pressure (RVSP, Fig. 1C). MiR‐339 expression level was significantly decreased in pulmonary arteries in MCT‐treated rats with PAH (Fig. 1E) compared to the control rats. In addition, PDGFR*β* was increased in MCT‐PAH rats (Fig. 1D), as previously reported (Jones et al. 2006). Together, the decrease in miR‐339 expression in pulmonary arteries of MCT‐PAH rats implicates its role in development of PAH.

### MiR‐339 expression can be downregulated by PDGF‐BB

**Figure 2:**
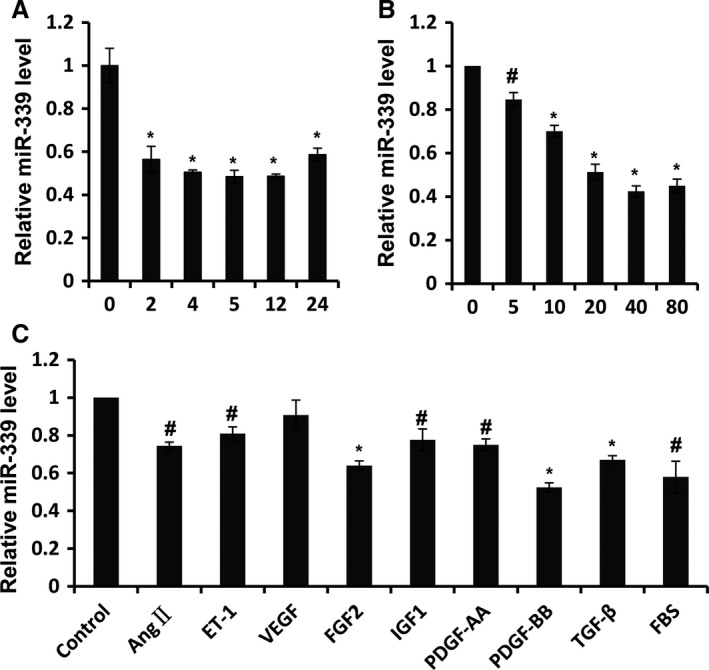
MiR‐339 was downregulated PDGF‐BB. (A) RPASMC was treated with PDGF‐BB for time indicated and qRT‐PCR was performed to assess the abundance of miR‐339, *n* = 3, *P < 0.01 versus 0 h. (B) RPASMC was exposed to PDGF‐BB with dose indicated for 12 h and qRT‐PCR was performed to detect the abundance of miR‐339, *n* = 3, #*P* < 0.05, ** *P* < 0.01 versus 0 ng/mL. (C) RPASMC was treated with cytokines or growth factors indicated for 12 h and the expression of miR‐339 was determined via qRT‐PCR,* n* = 3, #*P* < 0.05, *P < 0.01 versus control.

To further study the longitudinal expression of miR‐339, RPASMC were treated with PDGF‐BB for different time periods (0 h, 2 h, 4 h, 6 h, 12 h, and 24 h) and miR‐339 was measured by qRT‐PCR. As shown in Fig. 2A, expression of miR‐339 was significantly reduced by PDGF‐BB as early as 2 h poststimulation, and remained at low levels in later time points (Fig. 2A). In addition, PDGF‐BB treatment repressed expression of miR‐339 in a dose‐dependent manner (Fig. 2B). In addition to PDGF‐BB, some other cytokines and growth factors, such as transforming growth factor‐*β* (TGF*β*), fibroblast growth factor 2 (FGF2), insulin‐like growth factor‐1 (IGF1), angiotensin II (ANGII), and endothelin‐1 (ET‐1) participate in regulating proliferation of PASMC (Saltis et al. 1992; Bayes‐Genis et al. 2000; Millette et al. 2006; Berghausen et al. 2013; Leung et al. 2013). To determine whether miR‐339 responded universally to these cytokines and growth factors, miR‐339 was measured in RPASMC stimulated with ANGII, ET‐1, FGF2, IGF1, PDGF‐AA, PDGF‐BB, TGF*β*, VEGF, or fetal bovine serum (FBS) for 12 h, respectively. As the results indicate, miR‐339 was downregulated in response to most of the factors, except for VEGF (Fig. 2C).

### PDGF‐BB reduced miR‐339 expression through its effects on ERK and PI3K

**Figure 3:**
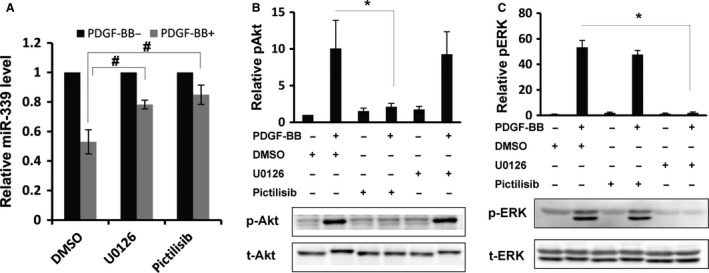
PDGF‐BB downregulated miR‐339 through PI3K and ERK activity. (A) RPASMC was pretreated for 30 min with U0126 or pictilisib, following exposure to PDGF‐BB for 12 h, expression of miR‐339 was determined by qRT‐PCR. *n* = 3, #*P* < 0.05, with Student's *t* test comparing indicated group. RPASMC was pretreated with U0126 or pictilisib for 30 min, following stimulation with PDGF‐BB for 5 min, western blot was performed to determine the protein level of pAkt, Akt (B) and pERK, ERK (C). *i* = 3, *P < 0.01 between indicated groups.

It is well known that PDGF‐BB regulates function of VSMC by activating distinct kinase activity (Kazlauskas 1994; Heldin and Westermark 1999; Rosenkranz and Kazlauskas 1999). To identify upstream mediators, miR‐339 expression was measured after selective kinases were inhibited with corresponding inhibitors. As our results show, downregulation of miR‐339 induced by PDGF‐BB was partly abrogated when PI3K activity was inhibited with pictilisib or when ERK activity was repressed by U0126 (Fig. 3A). However, inhibition of STAT and PKC activity with corresponding inhibitors had little effect on PDGF‐BB‐induced downregulation of miR‐339 (data not shown). Western blot was performed to confirm the inhibition of ERK (Fig. 3C) and PI3K (Fig. 3B) activation with their corresponding inhibitors. These data indicate that PDGF‐BB reduced miR‐339 expression through both ERK and PI3K.

### MiR‐339 inhibited the proliferation of PASMC

**Figure 4:**
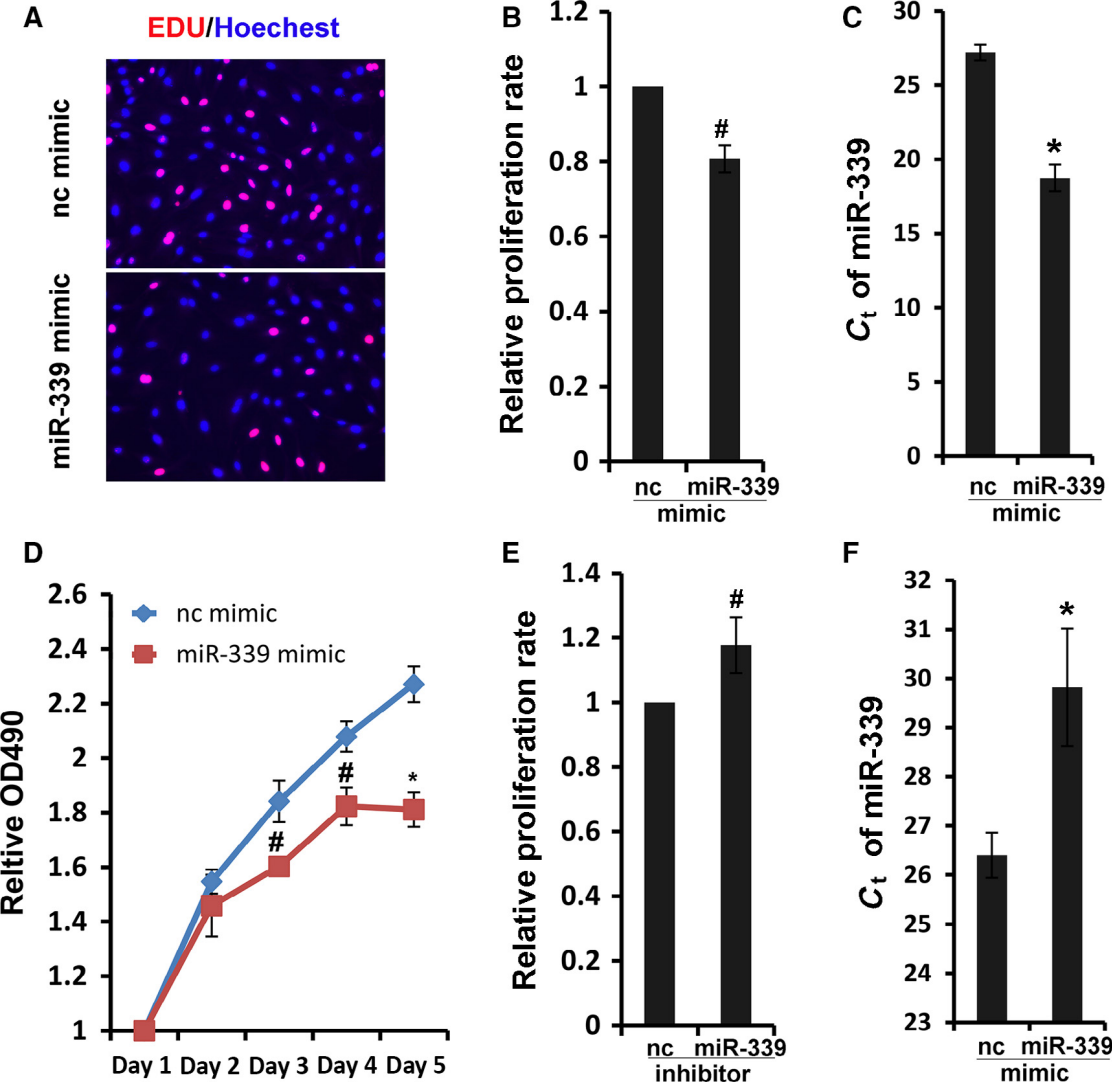
MiR‐339 inhibited RPASMC proliferation. (A) RPASMC transfected with miR‐339 or control mimic for 72 h were incubated with EdU solution for 4 h, and then were fixed and stained with Apollo dye (red) and Hoechest (blue). (B) Bar charts showing relative EdU incorporation rate in RPASMC,* n* = 4, #*P* < 0.05 versus control mimic. (C) Quantification of miR‐339 level was assessed in transfected RPASMC with qRT‐PCR, *P < 0.01 versus control mimic. (D) Proliferation of RPASMC infected with control or miR‐339 mimic was determined using MTS assay, *n* = 4, # *P* < 0.05, *P < 0.01 versus control mimic. (E) Proliferation of RPASMC infected with control or miR‐339 inhibitors was determined via EdU incorporation assay, *n* = 4, #*P* < 0.05 versus control mimic. (F) Quantification of miR‐339 level was determined in transfected RPASMC with qRT‐PCR *P < 0.01 versus control inhibitor.

To understand the biological function of miR‐339 downregulation, we investigated the effect of miR‐339 on the proliferation of RPASMC. EdU incorporation assay showed that miR‐339 mimic significantly suppressed proliferation of RPASMC compared to its control (Fig. 4A–C). MTS assay, a specific assay that could reflect actively proliferating cells through measuring the activity of cell metabolism, also showed less viable activity in miR‐339‐overexpressed cells compared to the mimic control (Fig. 4D). To further confirm the role of miR‐339 in PASMC proliferation, miR‐339 was repressed with an inhibitor and the effect on proliferation of RPASMC was assessed. As the results show, the miR‐339 inhibitor significantly increased the proliferation rate of RPASMC compared to its control (Fig. 4E and F). These results suggest that miR‐339 plays an important physiological role in proliferation of PASMC.

### FRS2 is a potential target of miR‐339 in PASMC

**Figure 5:**
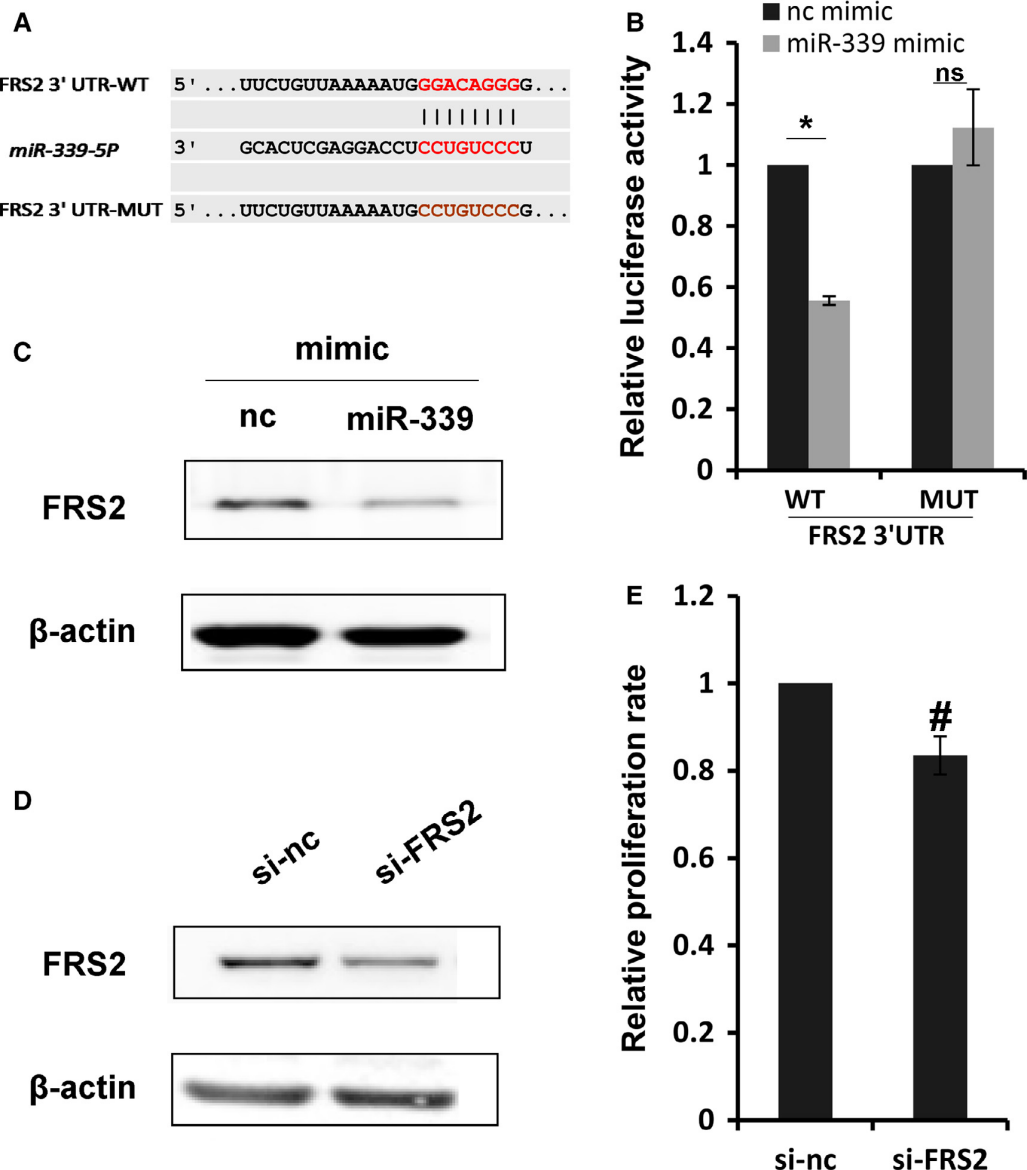
FRS2 is a potential target of miR‐339. (A) putative miR‐339 binding sites in the 3′ UTR of FRS2 along with the mutation sites. (B) 3′ UTR luciferase reporter constructs with wild‐type (WT) or mutant (MUT) sequence of FRS2 3′ UTR transfected with miR‐339 or control mimic. Bar charts of luciferase reporter analysis represent mean ± SE (*n* = 3), firefly luciferase activity was normalized to renilla luciferase activity, *P < 0.01, with Student's *t* test comparing indicated group, ns represents no significance. (C) RPASMC was transfected with miR‐339 or control mimic for 72 h, western blot was performed to determine the protein level of FRS2, *β*‐actin was used as endogenous control, *n* = 3. (D) RPASMC transfected with si‐FRS2 or control siRNA for 72 h, western blot was performed to determine the protein level of FRS2, *β*‐actin was used as endogenous control, *n* = 3. (E) Bar charts showing relative EdU incorporation rate in RPASMC transfected with si‐FRS2 or control siRNA for 72 h, *n* = 4, #*P* < 0.05 versus control siRNA.

To investigate the mechanism of miR‐339‐induced inhibition of proliferation of RPASMC and identify novel targets of miR‐339, the online tool Targetscan (http://www.targetscan.org/) was used to search for a putative binding site of miR‐339 and it was found in the 3′ UTR of FRS2 mRNA (Fig. 5A). Luciferase reporter assays with transfected 3′ UTR sequences of FRS2 along with miR‐339 or control mimic showed that luciferase activity was significantly repressed by miR‐339 overexpression (Fig. 5B). In contrast, mutation of miR‐339 binding site resulted in a complete loss of the inhibitory effect of miR‐339 on luciferase activity (Fig. 5B). Next, we studied the effect of miR‐339 mimic on the expression of endogenous FRS2. As expected, overexpression of miR‐339 caused a significant decrease in FRS2 protein level (Fig. 5C). To investigate whether FRS2 mediated the effects of miR‐339 on proliferation of RPASMC, we inhibited FRS2 with siRNA and then studied its effect on cell proliferation. As shown in Figure 5E, the proliferation rate of RPASMC was significantly decreased due to the loss of FRS2 (Fig. 5D and E). Overall, these results suggest that miR‐339 regulates RPASMC proliferation at least partially by targeting FRS2.

### MiR‐339 only represses FGF2‐induced proliferation of PASMC

**Figure 6:**
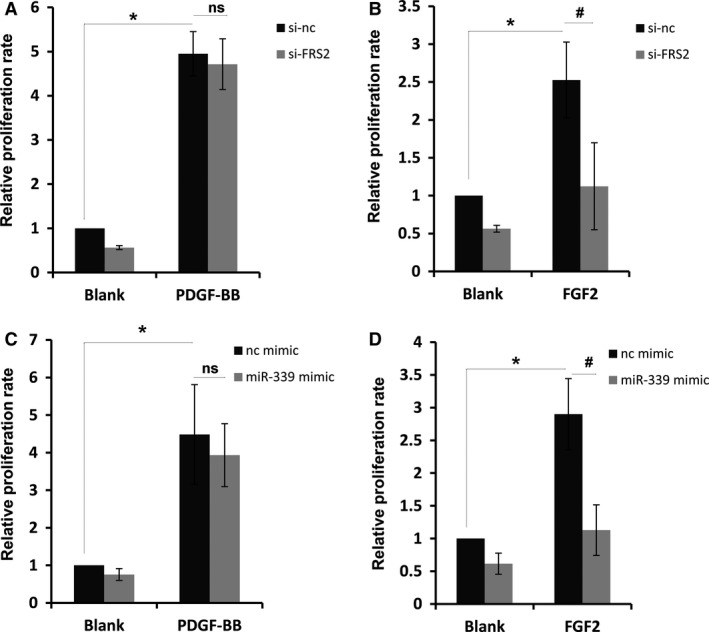
MiR‐339 repressed FGF2‐induced proliferation. RPASMC transfected with si‐FRS2 or control siRNA for 72 h was starved for 12 h, then PDGF‐BB (A) or FGF2 (B) was added for 12 h. Proliferation rate of RPASMC was determined with EdU incorporation assay. *n* = 3, #*P* < 0.05, *P < 0.01, comparing indicated group, ns represents no significance. RPASMC transfected with miR‐339 or control mimic for 72 h was starved for 12 h, then PDGF‐BB (C) or FGF2 (D) was added for 12 h. Proliferation rate of RPASMC was determined with EdU incorporation assay. *n* = 3, #*P* < 0.05, *P < 0.01, comparing indicated group, ns represents no significance.

FRS2 is an adaptor protein and plays a critical role in FGF signaling transduction (Chen et al. 2009). A previous report has shown that loss of FRS2 can inhibit proliferation of VSMC induced by FGF2, but not affect that induced by PDGF‐BB (Chen et al. 2009). We tried to confirm the role of FRS2 during these processes in PASMC. As the results show, both PDGF‐BB and FGF2 treatment sharply increased proliferation of PASMC, and loss of FRS2 significantly abrogated the positive effect of FGF2 on proliferation of PASMC but not that of PDGF‐BB (Fig. 6A and B). The effect of miR‐339 overexpression is consistent with the effect of FRS2 knockdown on proliferation of PASMC, since as expected, miR‐339 mimic significantly abrogated the positive effect of FGF2 on proliferation of PASMC but not that of PDGF‐BB (Fig. 6C and D). These results further confirm that miR‐339 represses proliferation of PASMC by targeting FRS2.

## Discussion

**Figure 7:**
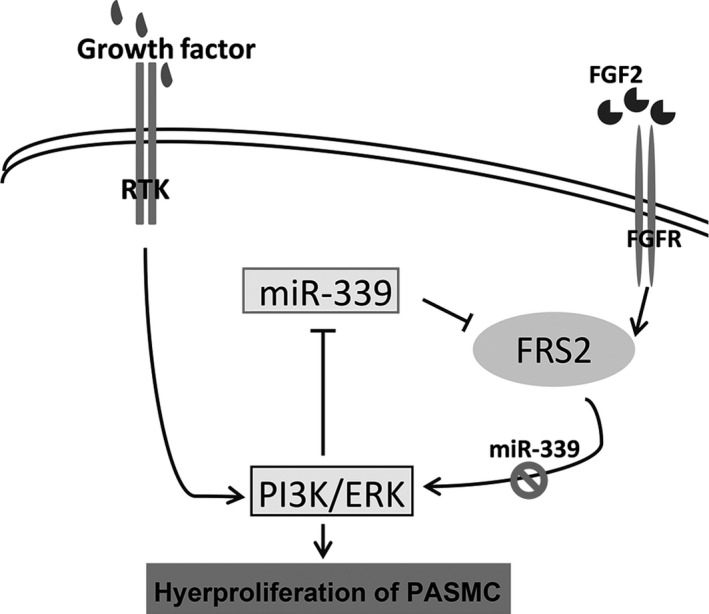
Schematic model illustrating the regulation and the functional roles of miR‐339 in PASMC. Binding to corresponding receptors, cytokines and growth factors activated PI3K and ERK, and then decreased the expression of miR‐339. Reduced miR‐339 promoted expression of FRS2, which caused activation of ERK and PI3K in a positive feedback manner.

Although many miRNAs have been reported in the biological regulation of cardiovascular cells (Barwari et al. 2016), the role of miRNAs in cardiovascular system is still being elucidated. In this study, we found that miR‐339 is highly expressed in the cardiovascular system and that it can be downregulated by a number of cytokines and growth factors, especially PDGF‐BB and FGF2, which is dependent on activation of ERK and PI3K. In functional studies, we found that miR‐339 repressed proliferation of PASMC by targeting FRS2, a key adaptor protein in FGF signaling transduction. Furthermore, miR‐339 expression was significantly decreased in the pulmonary arteries of MCT‐induced PAH rats, implicating its role in development of PAH (Fig. 7).

Essential pathological characteristics of PAH are excessive proliferation and migration of pulmonary arterial smooth muscle cell (PASMC), leading to medial hypertrophy and vascular remodeling (Morrell et al. 2009; Soubrier et al. 2013). In normal physiological state, PASMC are typically quiescent and contractile (Gomez and Owens 2012). However, PASMC phenotype can switch to a more synthetic and pro‐proliferative state in vascular injury and other pathological conditions that induce the release of cytokines and growth factors, such as platelet‐derived growth factor BB (PDGF‐BB), transforming growth factor‐*β* (TGF*β*), fibroblast growth factor 2 (FGF2), insulin‐like growth factor‐1 (IGF1), tumor necrosis factor‐*α* (TNF*α*), and interleukin‐1 (IL‐1) (Bayes‐Genis et al. 2000; Millette et al. 2006; Li et al. 2013). Some environmental factors also induce the phenotype switch of PASMC, such as hypoxia (Caruso et al. 2010). Among these factors, PDGF‐BB is the most potent mitogen for PASMC (Berghausen et al. 2013). miRNAs have been investigated extensively and found to play diverse roles during pulmonary arterial remodeling induced by PDGF‐BB (Berghausen et al. 2013). For example, Brandi and his colleagues cloned and sequenced miRNAs expressed in PASMC under vehicle‐ or PDGF‐BB‐treated conditions and found that miR‐221 was one of the few miRNAs enriched in PDGF‐BB‐treated PASMC, which acted as a modulator of the phenotypic change of PASMC via targeting c‐Kit and p27Kip1 (Davis et al. 2009). Li et al. (2013) performed miRNA microarray analysis in human aortic smooth muscle cell (SMC) stimulated with PDGF‐BB and identified miR‐638 as one of the most significantly downregulated miRNA in human VSMC in response to PDGF‐BB stimulation. In our previous report, miRNAs expression in PASMC exposed to PDGF‐BB was investigated globally for the first time and a group of miRNAs were found to be expressed differentially in response to PDGF‐BB stimulation (Chen et al. 2016; Qian et al. 2016). Among these miRNAs, miR‐328 was downregulated by PDGF‐BB, a miRNA that inhibited proliferation and migration of PASMC via targeting PIM‐1 (Qian et al. 2016), and miR‐376b expression was increased by PDGF‐BB, a miRNA that increased proliferation of PASMC via targeting BMPR2 (Chen et al. 2016). MiR‐339 is one of the differentially expressed miRNAs reported in our previous work (Chen et al. 2016). In this study, miR‐339 was found to be downregulated not only by PDGF‐BB, but also by many other cytokines and growth factors, including FGF2, ET‐1, ANG II, and so forth. Moreover, miR‐339 expression decreased significantly in the pulmonary arteries of MCT‐induced PAH rats. These data implicate a role for miR‐339 in PAH development.

Since vascular remodeling can be induced by many cytokines and growth factors, increased signaling caused by these factors may contribute to the pathobiology of PAH (Raines 2004; Grimminger and Schermuly 2010). For example, increased PDGF or PDGFR expression has been detected in lung tissue and small pulmonary arteries of experimental pulmonary artery hypertension (PAH) animals (Arcot et al. 1993; Cai et al. 1996; Kwapiszewska et al. 2005; Jones et al. 2006) and patients with PAH (Humbert et al. 1998; Lanner et al. 2005). FGF2 and its receptor levels were also found to be significantly increased in both PAH animal models and patients (Benisty et al. 2004; Izikki et al. 2009). Inhibition of both PDGF and FGF signaling have been shown to ameliorate PAH, however, treatment of patients with their inhibitors was accompanied by severe adverse events and drug discontinuation was common (Frost et al. 2015; Speich et al. 2015). Previous studies have focused on single pathways, and cross‐talk between different pathways needs more attention. MiRNAs could play an important role in connecting different pathways. For example, our previous report indicated a cross‐talk between PDGF and BMP signaling mediated by miR‐376b (Chen et al. 2016). In this work, we also discovered a cross‐talk between FGF signaling and other factors, including PDGF‐BB, mediated by miR‐339 via targeting FRS2.

FRS2 was originally discovered as a docking site for coordinated assembly of a multiprotein complex that includes GRB2, GAB1, and SOS1, and serves a critical role in the FGFR signaling pathway (Sato and Gotoh 2009). FRS2‐mediated signaling results in activation of Ras/mitogen‐activated protein kinase (MAPK) and PI3K/Akt signaling (Sato and Gotoh 2009). Our work indicates that miR‐339 downregulation, which was dependent on activation of ERK and PI3K, would increase the expression of FRS2. Hence, it is a positive feedback of PDGF signaling mediated by miR‐339, leading to signal amplification and greater remodeling in pulmonary arteries.

In conclusion, our findings demonstrate that miRNA‐339 plays a critical role in modulating proliferation of PASMC that is mediated through the FGF signaling pathway. Many kinds of cytokines and growth factors are tightly integrated into a core transcriptional network with FGF2, involved in smooth muscle proliferation, and miR‐339 functions as a critical switch in modulating smooth muscle proliferation and may be important in PAH pathogenesis.

## Conflict of Interest

No conflicts of interest are declared by the authors.
